# Efficacy of Kampo Medicine in Treating Atopic Dermatitis: An Overview

**DOI:** 10.1155/2013/260235

**Published:** 2013-12-29

**Authors:** Tadamichi Shimizu

**Affiliations:** Department of Dermatology, Graduate School of Medicine and Pharmaceutical Sciences, University of Toyama, 2630 Sugitani, Toyama 930-0194, Japan

## Abstract

Atopic dermatitis (AD) is a common inflammatory skin disease with recurring episodes of itching and a chronic relapsing course. Current treatment options for AD include topical agents, such as topical corticosteroids and oral antiallergic drugs. Providing effective long-term treatment is sometimes difficult due to the chronic, relapsing nature of AD; therefore, there is a need to identify better therapeutic options with minimal side effects that are well tolerated over the variable course of the disease. Traditional herbal medicine, also known as Kampo medicine in Japan, has a long history and plays a role in the prevention and treatment of various diseases, including AD. Some Kampo medicines are useful for treating inflammatory skin diseases, and there has been increased interest in using Kampo medicine to develop new therapeutic agents for AD. Standard Kampo formulas for AD are effective in removing the symptoms of “*Netsu Sho*,” “*Ketsu-Kyo*,” “*Ki-Kyo*,” and “*O-Ketsu*.” This paper discusses the efficacy of Kampo medicines in treating AD. Knowledge of the mechanisms of action of Kampo medicines will result in greater choices of pharmacotherapeutic agents for AD.

## 1. Introduction

Atopic dermatitis (AD), also known as atopic eczema, is a common chronic inflammatory skin disease characterized by infiltration of inflammatory cells, extensive pruritus, and a clinical course of symptomatic flares and remissions [1]. The pathogenesis of the disease is now better understood, and important factors involved in the process include genetic factors, skin barrier dysfunction, and immune dysregulation [2]. The lesions are often erythematous with edematous, weeping papules, and vesicles in patients with acute AD, while lichenified thickened plaques were observed in patients in the chronic stage of the disease. Current treatment options for AD comprise topical agents, such as topical corticosteroids and oral antiallergic drugs, to overcome inflammation and moisturizing agents to improve physiologic skin dysfunction. Some AD patients are refractory to these conventional treatments, and current AD therapeutic guidelines recommend the use of further intensive treatment options, such as ultraviolet phototherapy or oral cyclosporine [3]. However, providing effective long-term treatment is sometimes difficult due to the chronic, relapsing nature of AD; therefore, there is a need to identify better therapeutic options with minimal side effects that are well tolerated over the variable course of the disease.

Traditional herbal medicine, also known as Kampo medicine in Japan, has a long history and plays a role in the prevention and treatment of various inflammatory skin diseases, including AD. Indeed, appropriate therapy with Kampo herbal drugs has been proven to be effective in patients with AD who are resistant to basic treatment. Moreover, many patients currently visit physicians seeking Kampo medicine treatment.

## 2. Kampo Medicine for AD in Japan

Recently, the use of alternative and complementary medicines has become popular in Western countries, where Kampo medicines are now available. The use of Kampo medicines is evolving as a comprehensive approach to treatment and is combined with established Western medical drugs. Kampo medicine was originally introduced from China more than 1,500 years ago. Due to its unique development in Japan, it is quite different from traditional Chinese medicine, and Kampo prescriptions have been used to treat various diseases for centuries in Japan. To date, the Japanese Ministry of Health, Labour, and Welfare has approved 148 Kampo prescription products for use in clinical practice.

Western medicine is anchored in the treatment of the pathogenesis and pathological condition of a disease, whereas Kampo medicine emphasizes totality, in particular improving constitutional aspects. The concept of “*Sho*” originates from traditional Chinese medicine; however, it has been simplified as a result of the Kampo theory [4]. “*Sho*” refers to the pattern of symptoms present at any moment, and Kampo herbal drugs are usually prescribed according to the patient's “*Sho,*” such as “*Yin*” (negativity) and “*Yang*” (positivity) or “*Kyo*” (deficiency) and “*Jitsu*” (fullness), in order to target components, such as “*Ki*” (energy, spirit, and function), “*Ketsu*” (blood and organs), and “*Sui*” (fluid), all of which are considered to be basic components constituting the human body [5]. When treating a patient, Japanese practitioners recognize the Kampo diagnosis of “*Sho*” and choose the most suitable formula.

Some Kampo medicines are useful for treating inflammatory skin diseases, and there has been increased interest in using Kampo herbal drugs to develop new therapeutic agents for AD. In 2010, the report, “Evidence Reports of Kampo Treatment 2010—345 Randomized Controlled Trials—,” was published by the Committee for EBM, the Japan Society for Oriental Medicine (published on the website of the Japan Society for Oriental Medicine), promoting several Kampo medicine preparations as effective against AD. Standard Kampo formulas for AD are effective in removing the symptoms of “*Netsu Sho,*” “*Ketsu-Kyo,*” “*O-Ketsu*,” and “*Ki-Kyo*” (Table 1). Byakkokaninjinto and Oren-gedoku-to have been used to remove “*Netsu Sho*” symptoms, Tokii-inshi has been used to eliminate “*Ketsu-Kyo*” symptoms, and Keishibukuryogan and Kamisyoyosan have been used to clear away “*O-Ketsu*” symptoms. Hochu-ekki-to and Juzen-taiho-to have been used to remove “*Ki-Kyo*” symptoms, which include a delicate nature, easy fatigability, and/or hypersensitivity in AD patients [6, 7]. To date, several clinical and general studies have been reported in which these preparations were used to treat AD, and new evidence in support of Kampo medicine therapy has been obtained.

## 3. Kampo Formulas

### 3.1. Byakkokaninjinto

Byakkokaninjinto contains the extract of five medicinal plants (*Gypsum fibrosum*, *Anemarrhenae rhizoma*, *Glycyrrhizae radix*, *Ginseng radix,* and *Oryzae fructus*) and is commonly used to improve symptoms of “*Netsu Sho,*” which include dry mouth, hot flashes, perspiration, and pruritus. A recently published report showed that Byakkokaninjinto treatment increases the expression of aquaporin 5, which is known to regulate salivary secretion from the submandibular gland [8]. It has also been suggested that Byakkokaninjinto may improve dry mouth, such as that observed in patients with Sjögren syndrome, by increasing the expression of aquaporin 5 and enhancing salivary secretion [8].

Byakkokaninjinto is effective in improving facial pruritus and erythema in many cases of AD. Indeed, in patients with severe facial erythema, it has been reported that hot flashes are significantly improved for one to two weeks after the administration of this drug. Seki et al. previously reported that thermographic images of the face obtained before and after the administration of Byakkokaninjinto to treat symptoms of “*Netsu Sho*” helped to objectively select effective drugs in each case [9]. In order to make the use of Byakkokaninjinto more appropriate and effective, a daily log of conditions was created to investigate the subjective symptoms and effects of the drug [9]. Subsequently, Natsuaki investigated facial skin temperatures using thermography before and after the administration of Byakkokaninjinto. The authors measured the resting skin temperature of the face in 20 AD patients with “*Netsu Sho*” symptoms, including dry mouth, hot flashes, and pruritus, using thermography [10]. The skin temperatures in the AD patients were significantly higher than those observed in the healthy controls (*P* < 0.01). The authors then examined the cooling effect of Byakkokaninjinto in the patients with AD and found that the oral administration of Byakkokaninjinto lowered the facial skin temperature after 90 minutes in the AD patients. Furthermore, the skin symptoms of hot flashes and pruritus were reduced in the AD patients with “*Netsu Sho.*” The results suggested that thermography is useful for evaluating the cooling effect of Byakkokaninjinto.

### 3.2. Keishibukuryogan

Keishibukuryogan is a traditional herbal medicine that is widely administered to patients with symptoms of “*O-Ketsu,*” which involves blood stasis, in order to improve blood circulation. Matsumoto et al. explored the use of a proteomic approach for diagnosing blood stasis in rheumatoid arthritis patients treated with Keishibukuryogan [11]. In addition, Keishibukuryogan is used to treat symptoms of peripheral ischemia, such as cold extremities [12]. Keishibukuryogan is now one of the most frequently used traditional medicines in Japan and has been used clinically to treat various diseases, including skin diseases.

Keishibukuryogan preparations demonstrate anti-inflammatory and free radical scavenging effects. Keishibukuryogan is composed of five medicinal plants (*Cinnamomi cortex*, *Paeoniae Radix*, *Moutan cortex*, *Persicae semen,* and *Hoelen*) [13]. *Paeoniae Radix* and *Moutan Cortex* contain many known active components that are common in both plants, including paeoniflorin, paeonol, oxypaeoniflorin, benzoylpaeoniflorin, and palbinone [14]. Paeoniflorin is a characteristic principal bioactive component of *Paeoniae Radix*, which contains approximately 5.57% (w/w) paeoniflorin, and *Moutan Cortex*, which contains approximately 3.96% (w/w) paeoniflorin [15]. Paeoniflorin has many pharmacological effects, including anti-inflammatory and antiallergic effects [16]. Keishibukuryogan and paeoniflorin suppress the production of macrophage migration inhibitory factor, interleukin (IL)-6, IL-8, and tumor necrosis factor-*α* in LPS-stimulated human dermal microvessel endothelial cells and prominent cells in the dermal skin [17]. Accordingly, Keishibukuryogan may have beneficial effects that result in the inhibition of inflammatory cytokines in human dermal microvessel endothelial cells.

Keishibukuryogan improves the conjunctional microcirculation in patients with cerebrospinal vascular diseases [18], thus suggesting that it may have beneficial effects on hematological parameters, such as blood viscosity and red blood cell deformability [19, 20]. In addition, Keishibukuryogan exerts useful effects on the endothelial function in patients with metabolic syndrome-related factors [21]. AD lesions are characterized by differences in the state of activation of endothelial cells and the release of inflammatory mediators by and toward the vasculature [22]. Longstanding inflammatory skin conditions result in itching-induced scratching, which causes cutaneous damage, including the manifestation of endothelial cells as lichenification. Recently, the long-term administration of Keishibukuryogan was found to achieve marked improvements in patients with a high level of lichenification [23]. Consequently, it is believed that such long-term administration is effective in patients who exhibit a tendency toward the remission of symptoms due to Keishibukuryogan administration for approximately one month [23]. Therefore, Keishibukuryogan has been found to be effective against AD, particularly in patients with lichenified lesions, and may become a useful therapy for intractable AD in patients previously treated with conventional modalities.

### 3.3. Hochu-Ekki-to

Hochu-ekki-to contains 10 medicinal plants (*Astragali Radix*, *Atractylodis Lanceae Rhizoma*, *Ginseng Radix*, *Angelicae Radix*, *Bupleuri Radix*, *Zizyphi Fructus*, *Aurantii Nobilis Pericarpium*, *Glycyrrhizae Radix*, *Cimicifugae Rhizoma,* and *Zingiberis Rhizoma*). Hochu-ekki-to has been used traditionally to treat weak patients with chronic diseases possessing the symptoms of “*Ki-Kyo*,” which include loss of appetite, a mild fever, night sweats, palpitations, fear, restlessness, a weak and feeble voice, slurred speech, and a disturbance of vision [24]. This medicine is known to have an effect in easily improving infectious conditions and recovering the body's protective capacity by enhancing the immune function and is intended to be applied in patients exhibiting symptoms in the respiratory apparatus [25], given its clinical efficacy in treating chronic colds and preventing respiratory infection with MRSA in patients with impaired consciousness. The oral administration of Hochu-ekki-to in mice succeeds in enhancing antigen-specific antibody responses in the systemic immune system via the upper respiratory mucosal immune system [24]. Furthermore, Hochu-ekki-to has various immunopharmacological effects, particularly antiallergic properties, including suppressing the serum IgE level and eosinophil infiltration and improving dermatitis by controlling the Th1/Th2 balance, possibly by inducing interferon-*γ* production from intraepithelial lymphocytes [26–28].

Previous case reports have suggested that Hochu-ekki-to is effective in a certain subgroup of patients with AD [29]. Following the administration of Hochu-ekki-to in patients with recalcitrant AD, the levels of eosinophils were statistically decreased after three months of treatment with this formula. The serum IgE levels showed a tendency to decrease following the administration of this substance [29].

The use of Chinese herbal medicine therapy in clinical studies has various restrictions due to the difficulty of creating placebos. A recent double-blind, placebo-controlled study showed considerably effective benefits in managing the clinical signs of AD with Hochu-ekki-to [30]. In that study, 91 AD patients with “*Ki-Kyo*” symptoms were enrolled. Hochu-ekki-to or a placebo was orally administered twice daily for 24 weeks. The results showed that the total equivalent amount of topical agents, including topical steroids and/or tacrolimus, was significantly lower in the Hochu-ekki-to group than in the placebo group after the 24-week treatment period (*P* < 0.05), although the overall skin severity scores were not statistically different. That study demonstrated that Hochu-ekki-to is a useful adjunct to conventional treatments in AD patients with a “*Ki-Kyo*” constitution. The use of Hochuekki-to significantly reduces the dose of topical steroids and/or tacrolimus in AD patients without aggravating AD. Recently, Takemura et al. conducted a clinical study in which Hochu-ekki-to was used in combination with topical corticosteroids in AD patients [31]. Consequently, a significant decrease in the use of topical steroids was observed.

### 3.4. Juzen-Taiho-to

Juzen-taiho-to consists of 10 different herbs (*Astragali Radix*, *Cinnamomi Cortex*, *Rehmanniae Radix*, *Paeoniae Radix*, *Cnidii Rhizoma*, *Atractylodis Lanceae Rhizoma*, *Angelicae Radix*, *Ginseng Radix*, *Hoelen,* and *Glycyrrhizae Radix*) and has traditionally been used to treat symptoms of “*Ki-Kyo*,” which include fatigue, loss of appetite and anorexia, in patients with various diseases in Japan. At present, Juzen-taiho-to is often clinically used to treat some cancers and prevent adverse effects resulting from chemotherapy and radiation therapy, blood diseases, skin diseases, and so on [32]. Juzen-taiho-to suppresses primary melanocytic tumors by potentiating T-cell-mediated antitumor cytotoxic immunity *in vivo* [33]. AD patients successfully treated with Juzen-taiho-to have been reported in Japan [34, 35]. It has been suggested that the effects on Th1 responses achieved via innate immune signaling are one possible mechanism underlying the activity of Juzen-taiho-to in controlling morbid states of the skin in patients with AD [34].

## 4. Conclusion

Many Kampo medicines have anti-inflammatory and antiallergic effects. Several studies of the pharmacological effects of Kampo medicines and their constituent herbs have been conducted in recent years, and much evidence has been accumulated. The mechanisms of action of the herbs included in Kampo medicines are becoming clearer, in addition to the fact that these mechanisms are not found in Western medicines. Furthermore, the concept of “*Sho*” can be scientifically analyzed and is thus expected to become another accepted concept in Western countries. On the other hand, although considerable attention has been paid to traditional herbal medicines as a treatment option for AD, only a few reports have examined the efficacy of these medicines in a randomized, double-blind manner [30, 36, 37]. It was recently reported that herbal medicine significantly improves symptom severity and is well tolerated in patients with AD. However, the poor quality of the studies does not allow for valid conclusions to be drawn supporting the tolerability or routine use of these drugs [38].

Kampo medicines are now widely used worldwide. The general public tends to believe that these agents are safe because of their natural origin; thus, they are used frequently. However, administration of Kampo medicines has been reported to be associated with diverse side effects, such as interstitial pneumonia [39] and skin eruption [40]. We also need to pay attention to these side effects.

Additional studies addressing these methodological issues are warranted to determine the therapeutic benefits of Kampo medicine for AD.

## Figures and Tables

**Table 1:** Kampo formulas for atopic dermatitis.

Kampo formula	*Sho* (pattern of symptoms)	Skin symptoms
Byakkokaninjinto Oren-gedoku-to	*Netsu Sho* (dry mouth, hot flash, and perspiration)	Pruritus, erythema
Unsei-in	*Netsu Sho + Ketsu-Kyo* (dry mouth, hot flash, perspiration + pallor, and dizziness)	Pruritus, erythema, xerosis
Tokii-inshi	*Ketsu-Kyo* (pallor, dizziness)	Xerosis
Keishibukuryogan Kamisyoyosan	*O-Ketsu* (blood stasis/stagnant)	Lichenification
Hochu-ekki-to Juzen-taiho-to	*Ki-Kyo* (fatigue, drowsiness, or appetite loss)	(Fatigue, drowsiness)

## Abbreviations

AD: Atopic dermatitis
IL: Interleukin.
